# Urinary neutrophil gelatinase-associated lipocalin: a key biomarker for differentiating acute kidney injury and predicting short-term mortality in liver cirrhosis patients

**DOI:** 10.1080/0886022X.2026.2624277

**Published:** 2026-07-06

**Authors:** Pooja Basthi Mohan, Shankar Prasad Nagaraju, Balaji Musunuri, Ganesh Bhat, Ravindra Prabhu, Ravindra Maradi, Shiran Shetty

**Affiliations:** ^a^ Department of Gastroenterology and Hepatology, Kasturba Medical College, Manipal, Manipal Academy of Higher Education, Manipal, Karnataka, India; ^b^ Department of Nephrology, Kasturba Medical College, Manipal, Manipal Academy of Higher Education, Manipal Karnataka, India; ^c^ Department of Biochemistry, Kasturba Medical College, Manipal, Manipal Academy of Higher Education, Manipal, Karnataka, India

**Keywords:** Urinary NGAL, acute kidney injury, neutrophil gelatinase-associated lipocalin, biomarkers, hepatorenal syndrome, chronic liver disease

## Abstract

**Background:**

urinary neutrophil gelatinase-associated lipocalin (uNGAL) has emerged as a promising biomarker in clinical settings for the differential diagnosis of acute kidney injury (AKI) in chronic liver disease (CLD). This prospective study aims to evaluate the efficiency of uNGAL in the differential diagnosis of AKI among patients with CLD and in predicting short-term mortality in this population.

**Methods:**

This prospective observational study was conducted from September 2021 to March 2024 at Kasturba Medical College, Manipal, among patients with AKI and CLD. The study was initiated after obtaining institutional ethics committee approval. Demographic and clinical data, including urine samples for NGAL analysis, were collected. Patients were followed for 90 days to record clinical outcomes.

**Results:**

Among the 188 CLD patients, 42.6% (*N* = 80) exhibited prerenal AKI, 41.5% (*N* = 78) were diagnosed with hepatorenal syndrome (HRS), and 16% (*n* = 30) were identified as having acute tubular necrosis (ATN). u-NGAL Cutoffs of 631 ng/ml distinguish HRS from ATN [AUC: 0.949, 95% C.I (0.895-1)], with a sensitivity of 92% and specificity of 93%, while 257 ng/ml and 236 ng/ml predict 30-day [AUC: 0.807, 95% C.I (0.740–0.875)] and 90-day [AUC: 0.809, 95% C.I (0.745–0.873)] mortality, respectively. Elevated alanine transaminase (ALT), Model for End-stage Liver Disease-sodium (MELD-Na), and uNGAL levels serve as independent predictors of mortality. Patients with uNGAL >236 ng/ml show higher 90-day mortality, underscoring uNGAL’s diagnostic, and prognostic value in AKI.

**Conclusion:**

This study shows that uNGAL distinguishes HRS from ATN and serves as a valuable predictor of 30-day and 90-day mortality. Additionally, higher levels of ALT, MELD-Na score, and uNGAL were identified as independent risk factors for mortality.

## Introduction

Acute kidney injury (AKI) is a frequent complication in patients with chronic liver disease (CLD), with an approximate prevalence ranging from 20% to 50% and is associated with a markedly high mortality rate [1,2]. Cirrhosis is a complex condition marked by significant morbidity resulting from profound volume shifts and widespread vasodilation. The development of AKI adds further complexity to the clinical picture and should be carefully considered when evaluating a patient for liver transplantation [3,4].

AKI in cirrhosis is defined and staged by relative changes in serum creatinine (sCr) [5]. Higher AKI stages (more severe injury), correlate with higher mortality rates. Particularly, dialysis-dependent AKI, with over 80% mortality, signifies the poorest short-term prognosis [6,7]. Overall, the risk of mortality associated with AKI in cirrhosis is influenced by multiple factors, including the underlying cause and severity of AKI, the extent of liver dysfunction, and the presence of additional complications related to liver disease [8,9]. Therefore, timely and accurate diagnosis of AKI is crucial to enable early therapeutic intervention, which can significantly improve the chances of reversing renal dysfunction [10].

Prior to 2015, the diagnosis of AKI in cirrhotic patients relied on an arbitrary serum creatinine (sCr) threshold of ≥1.5 mg/dL. This lack of standardized diagnostic criteria led to considerable inconsistencies in identifying AKI, contributing to wide variability in its reported incidence among patients with cirrhosis [11]. The International Club of Ascites (ICA) updated the diagnostic criteria for AKI in cirrhosis by eliminating the traditional requirement of a serum creatinine (sCr) ≥1.5 mg/dL (133 µmol/L), aiming for a more sensitive and standardized definition [5].

The revised criteria also classify AKI into 3 stages based on the severity of sCr elevation. Stage 1 is defined by an increase in sCr of ≥0.3 mg/dL or 1.5 to 2 times the baseline value. Stage 2 is characterized by a 2 to 3-fold increase from baseline, while Stage 3 involves a rise of more than 3 times the baseline or the initiation of renal replacement therapy. This staging system facilitates risk stratification and guides timely therapeutic decisions in patients with cirrhosis [5,12].

AKI may arise from prerenal, intrinsic renal, or postrenal causes. Prerenal AKI (PRA) occurs due to reduced renal perfusion without direct damage to the glomeruli or tubules [13,14]. If not promptly managed, it can progress to acute tubular necrosis (ATN), a form of intrinsic renal injury [15]. Notably, patients with cirrhosis present a distinct scenario, as they are also susceptible to developing hepatorenal syndrome (HRS), a unique type of functional renal failure associated with CLD [16,17].

The management of AKI in cirrhotic patients is largely guided by identifying the underlying cause. HRS is typically managed with pharmacological therapy, while PRA responds well to plasma volume expansion. However, this approach is not appropriate for ATN, which requires a different management strategy [9,18]. Unfortunately, distinguishing between the different types of AKI in cirrhotic patients is clinically challenging, as sCr lacks sufficient sensitivity and specificity to effectively differentiate among PRA, ATN, and HRS [19,20].

While no single diagnostic test is currently available to make this distinction reliably, the latest guidelines from the ICA emphasize the need for further research into urinary biomarkers. Among the various novel markers identified over the past decade, urinary neutrophil gelatinase-associated lipocalin (uNGAL) has shown the most promise in addressing this critical diagnostic gap [21,22]. NGAL is a small protein released from neutrophils and renal tubular cells in response to injury. It appears in plasma and urine within 2 h of kidney insult, significantly earlier than the rise in serum creatinine, which typically occurs 24–48 h later. uNGAL in particular has gained attention not only for differentiating types of AKI but also as a predictor of mortality in cirrhotic patients, often in combination with the MELD (Model for End-stage Liver Disease) score. Its high sensitivity and specificity make uNGAL a promising tool for early detection, risk stratification, and management of AKI in cirrhosis [21].

Therefore, this study was conducted to evaluate the efficiency of uNGAL in differentiating AKI among cirrhotic patients and predicting short-term mortality in this population. Additionally, the study analyzed independent risk factors contributing to mortality in AKI patients with liver cirrhosis.

## Methodology

**Figure 1.**
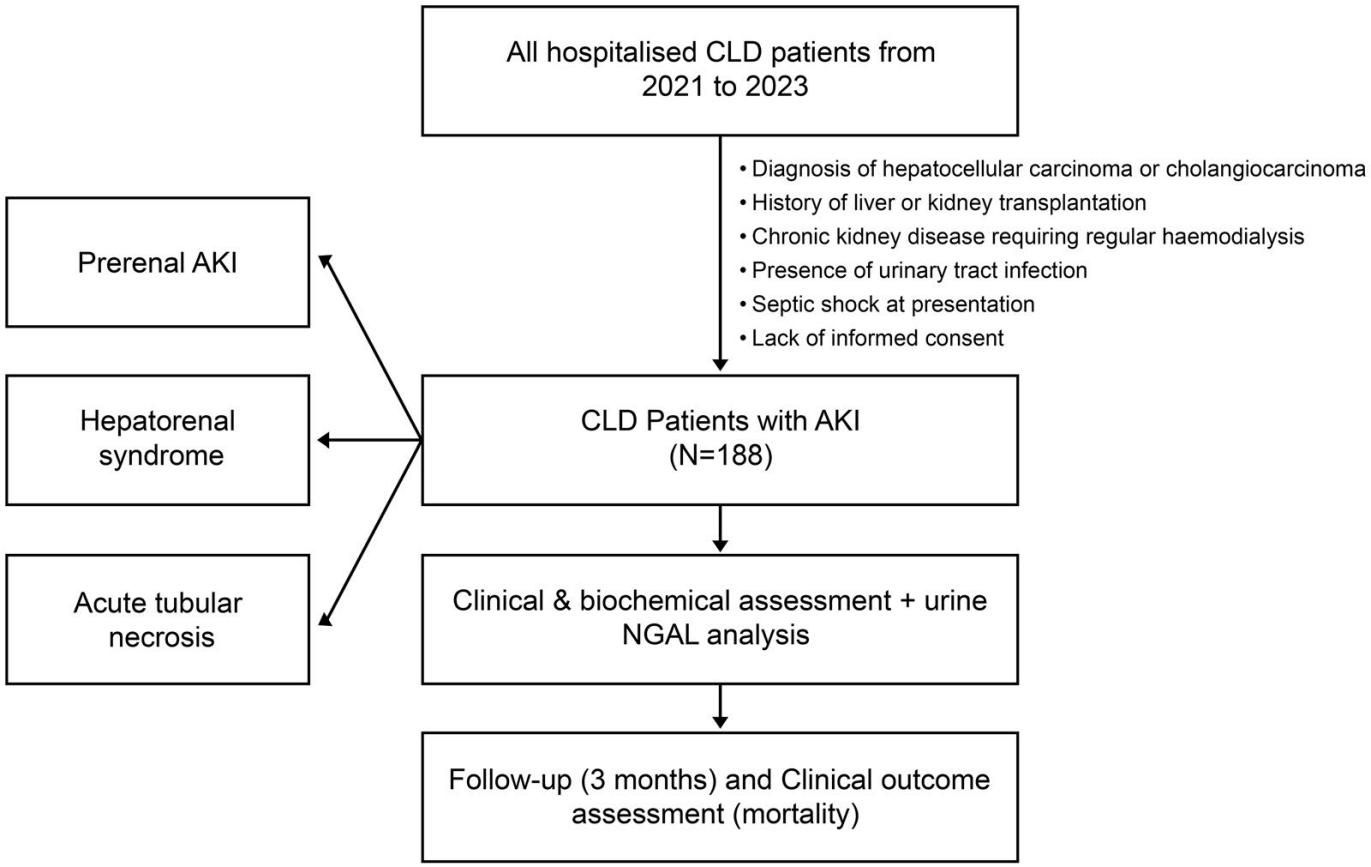
Flowchart representing the study methodology.

This prospective observational study was performed among patients with AKI in liver cirrhosis who attended the Department of Gastroenterology and Hepatology, Kasturba Medical College, Manipal, Karnataka, India from September 2021 to March 2024. Patients aged above 18 years with a confirmed diagnosis of liver cirrhosis and AKI were included in the study. Both patients presenting with AKI at admission and those who developed AKI during hospitalization were recruited, provided they met the diagnostic criteria. Ethical clearance for the study was granted by the Institutional Ethics Committee of Kasturba Medical College and Kasturba Hospital (IEC 338–2021), and the trial was registered with the Clinical Trials Registry of India (CTRI/2021/09/036171). Written informed consent was obtained from all the patients prior to enrollment. The study was conducted in accordance with the ethical principles of the Declaration of Helsinki and the Good Clinical Practice (GCP) guidelines. The exclusion criteria for the study included patients diagnosed with hepatocellular carcinoma or cholangiocarcinoma, individuals who had undergone liver or kidney transplantation, and those with chronic kidney disease maintained on regular hemodialysis prior to admission. Additionally, patients presenting with urinary tract infections, septic shock, or those who did not provide informed consent (Figure 1).

**Figure 2.**
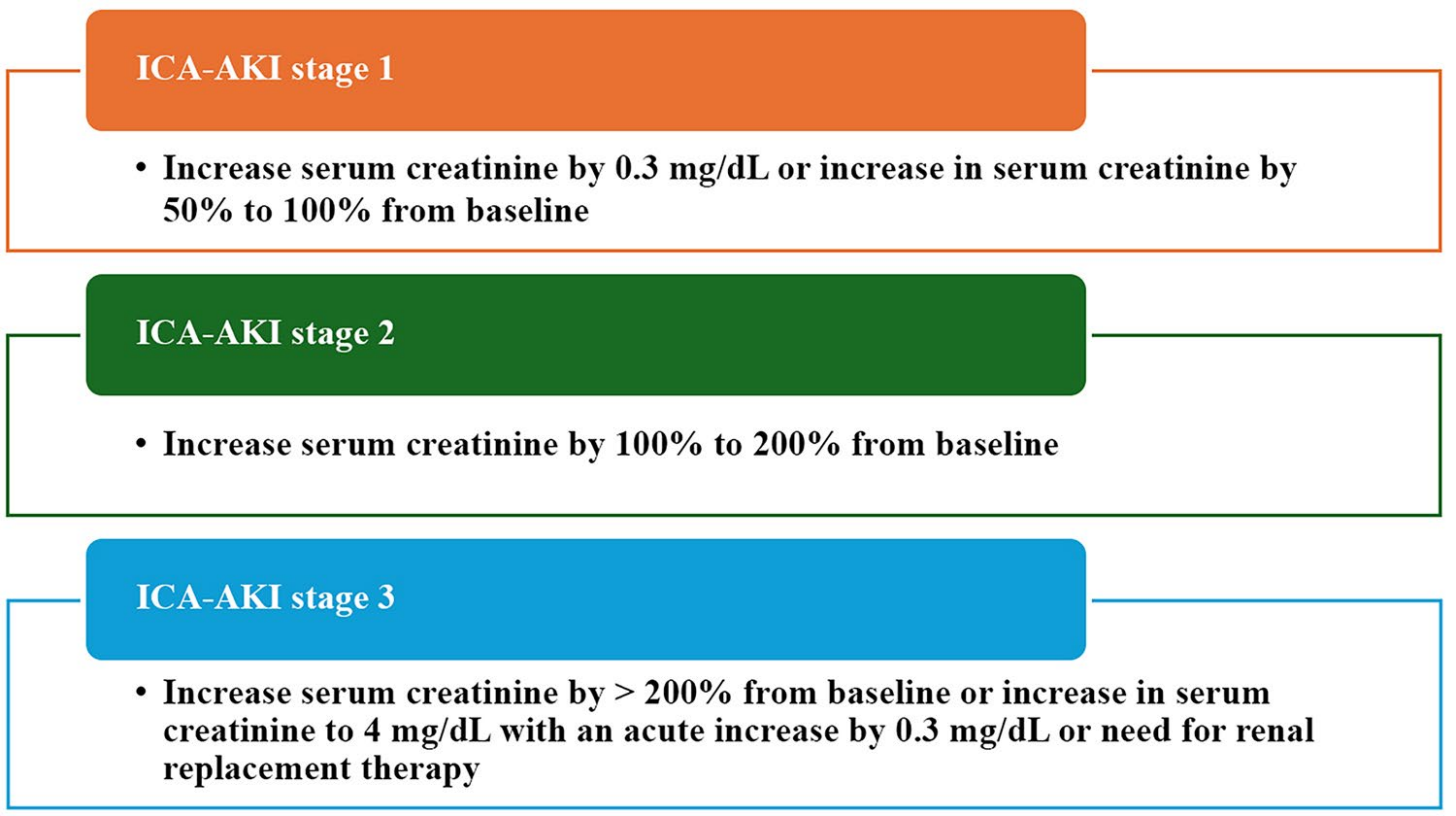
AKI staging.

Liver cirrhosis was diagnosed based on a combination of clinical presentation, biochemical markers, radiological findings, or histopathologic confirmation showing F4 fibrosis changes where available. AKI was identified using the ICA criteria, defined as an increase in sCr of more than 0.3 mg/dL within 48 h or a rise of ≥50% from the baseline value within the preceding 7 days. Baseline creatinine was considered as the most recent stable value recorded within 3 months prior to admission; if unavailable, the admission creatinine value was used as the baseline. Following diagnosis, patients were classified and staged according to the standardized staging criteria for AKI [5] (Figure 2).

### Classification of AKI

Participants were categorized into one of three types of AKI: PRA, HRS, or ATN. The diagnosis of HRS was established according to the 2015 ICA criteria. Chronic liver disease with increase in sCr ≥0.3 mg/dl within 48 h or ≥50% from baseline and/or Urinary output ≤0.5 mL/kg B.W. ≥6 h, no complete or partial response was observed after at least two days of discontinuing diuretics and administering volume expansion with albumin, absence of shock, no current or recent treatment with nephrotoxic drugs, absence of parenchymal disease [5,23].

Pre-renal AKI: Participants with a clinical history suggestive of a prerenal state such as gastrointestinal fluid loss or bleeding who demonstrated improvement in serum creatinine levels after volume resuscitation and discontinuation of diuretics were classified as having PRA. ATN was diagnosed in participants who did not fulfill the criteria for either PRA or HRS. This diagnosis was based on a clinical history indicative of tubular/parenchymal kidney injury, and no response to treatment within 2 days of volume replacement, with or without a urinalysis revealing granular casts [24].

Demographic details, underlying etiology, relevant laboratory investigations, and any episodes of decompensation were systematically recorded using a predefined data collection form. The severity of liver disease was assessed using both the Child-Turcotte-Pugh (CTP) score and the Model for End-Stage Liver Disease (MELD) score.

Daily samples of serum, plasma, and urine were collected to generate aliquots for routine biochemical analyses. For uNGAL estimation, a 5 mL fresh urine sample was collected on the first day of admission, centrifuged at 1500 rpm, and the resulting supernatant was stored at −80 °C. It was then measured using a Fine Test ELISA kit (Cat. No. EH0012), following the manufacturer’s protocol. All samples were tested in duplicate, and the results were reported in nanograms per milliliter (ng/mL).

### Follow-up and outcome assessment

All patients underwent treatment following standardized protocols and was monitored for a period of 90 days. The follow-up period for all patients extended to 90 days, during which clinical outcomes were evaluated regarding mortality at both the 30-day and 90-day intervals.

### Sample size calculation

Anticipating a sensitivity of 80% for uNGAL as a biomarker with a precision of 10% at 95% confidence level a minimum of 188 liver cirrhosis patients AKI were recruited in the study.

$$N=4pq/d^{2}/\text{positivity rate on screening}$$

### Statistical analysis

Continuous variables were reported as mean ± standard deviation (SD), while categorical variables were expressed as percentages (%). Differences in normally distributed continuous variables were assessed using Analysis of Variance (ANOVA) or the Kruskal–Walli’s test, as appropriate, and categorical variables were compared using the Chi-square test. For multiple group comparisons based on AKI types, ANOVA was followed by Tukey’s *post hoc* test.

The diagnostic utility of uNGAL for differentiating AKI types was evaluated using the area under the receiver operating characteristic (AUROC) curve, which also helped determine the most sensitive and specific cutoff values. Similarly, AUROC analysis was employed to identify optimal cutoff values of uNGAL for predicting 30-day and 90-day mortality. The optimal uNGAL threshold obtained from ROC analysis was further used for survival analysis *via* the Kaplan–Meier method. A p-value of <0.05 was considered statistically significant.

## Results

A total of 188 patients were recruited with the mean age of 52.69 ± 12.14 years. 91.5% (*n* = 172) of the study cohort were male. Regarding Comorbidities, 31.4% (*n* = 59) had diabetes and 14.9% (*n* = 28) had hypertension. The primary causes of cirrhosis included alcohol, affecting 78.2% (*n* = 147) of the overall population, followed by nonalcoholic fatty liver disease (NAFLD) at 17% (*n* = 32), and hepatitis B/C accounting for 5.3% (*n* = 10).

Regarding the classification of AKI based on their presentations, 42.6% (*N* = 80) exhibited prerenal AKI, 41.5% (*N* = 78) were diagnosed with HRS, and 16% (*n* = 30) were identified as having ATN. AKI staging indicated that 52.1% (*n* = 98) were categorized as AKI stage 1, 39.9% (*n* = 75) as AKI stage 2, and only 8% (*n* = 15) as AKI stage 3. The severity scores for the diseases, MELD, MELD Na, and CTP, exhibited a significant increase in the ATN (25.43 ± 6.27, 26.30 ± 4.69, 10.93 ± 1.59) group, with subsequent elevations observed in the HRS (24.42 ± 7.22, 25.45 ± 7.57, 10.18 ± 1.93) and PRA groups (19.90 ± 5.59, 20.72 ± 5.76, 9.23 ± 1.43) (*p* = 0.001). Among the recruited population, 52.7% were categorized under the CTP C group, with HRS accounting for 45.5% (45 individuals), a statistically significant proportion compared to both the ATN and PRA groups (*p* = 0.002) (Table 1).

**Table 1.** Demographic and baseline clinical characteristics of liver cirrhosis patients with AKI.

Parameters	Whole cohort (*n* = 188)	Prerenal AKI (*n* = 80)	Hepatorenal syndrome (*n* = 78)	Acute tubular necrosis (*n* = 30)	*p*-value
Age (Mean ± SD)	52.69 ± 12.14	52.56 ± 11.8	53.72 ± 11.85	50.33 ± 13.78	0.430
Sex, % (N)	91.5% (172)	41.3% (71)	41.3% (71)	17.4% (30)	0.167
BMI (kg/m^2^)	26.09 ± 4.505	26.47 ± 4.16	25.70 ± 4.91	26.08 ± 4.31	0.561
Diabetes, % (N)	31.4% (59)	45.8% (27)	49.2% (29)	5.1% (3)	0.020*
Hypertension, % (N)	14.9% (28)	35.7% (10)	60.7% (17)	3.6% (1)	0.040
Etiology, % (N)					
Ethanol	78.2% (147)	38.1% (56)	43.5% (64)	18.4% (27)	0.043
NAFLD	17% (32)	53.1% (17)	37.5% (12)	9.4% (3)	0.332
HCV/HBV	5.3% (10)	40% (4)	50% (5)	10% (10)	0.804
Autoimmune/Budd Chiari	3.7% (7)	71.4% (5)	28.6% (2)	0% (0)	0.237
AKI stage,					0.045*
1	52.12% (98)	52% (51)	37.8% (37)	10.2% (10)	
2	39.89% (75)	33.3% (25)	45.3% (34)	21.3% (16)	
3	15	26.7% (4)	46.7% (7)	26.7% (4)	
MELD (Mean ± SD)	22.66 ± 6.83	19.90 ± 5.59	24.42 ± 7.22	25.43 ± 6.27	p = 0.001
MELD-Na (Mean ± SD)	23.60 ± 6.88	20.72 ± 5.76	25.45 ± 7.57	26.30 ± 4.69	0.001*
CTP (Mean ± SD)	9.90 ± 1.79	9.23 ± 1.43	10.18 ± 1.93	10.93 ± 1.59	0.001*
CTP (Mean ± SD)					0.002*
B	45.7% (86)	53.5% (46)	38.4% (33)	8.1% (7)	
C	52.7% (99)	31.3% (31)	45.5% (45)	23.2% (23)	

*Significant.

Note: All the demographic and clinical values are from the first day of AKI diagnosis.

Abbreviations: AKI: Acute kidney disease; *BMI*: Basal metabolic Index; CTP: Child-Turcotte-Pugh; HBV/HCV: hepatitis B/C virus; INR: International Normalized Ratio; MELD: Model for End-stage Liver Disease; MELD-Na: Model for End-stage Liver Disease sodium; NAFLD: nonalcoholic fatty liver disease.

Laboratory investigations of all the recruited AKI patients revealed no significant differences in baseline parameters between the three groups (Table 2). However, the ATN group exhibited notably elevated aspartate aminotransferase (AST) levels (102.87 ± 58.883) (*p* = 0.020), creatinine levels (1.83 ± 0.874) (*p* = 0.018), and decreased albumin levels (2.55 ± 0.531) (*p* = 0.006). Similarly with complications related to liver cirrhosis. Nonetheless, Hepatic encephalopathy was prevalent in 52.2% of individuals with HRS (*p* = 0.002).

**Table 2.** Complications and laboratory parameters.

Characteristics	Prerenal AKI(*n* = 80)	Hepatorenal syndrome(*n* = 78)	Acute tubular necrosis(*n* = 30)	*p*-value
**Complications**				
Oliguria, % (N)	27.9% (12)	53.5% (23)	18.6% (8)	0.091
Ascites, % (N)	42.5% (71)	41.9% (70)	15.6% (26)	0.901
HE, % (N)	26.1% (18)	52.2% (36)	21.7% (15)	0.002*
SBP, % (N)	48.4% (15)	38.7% (12)	12.9% (4)	0.747
EV,	38.9% (35)	41.1% (37)	20% (18)	0.339
Small EV Large EV	51.7% (30)	36.2% (21)	12.1% (7)	
**LFT (**Mean ± SD)				
Total Bilirubin (mg/dL)	4.54 ± 2.698	4.98 ± 2.948	5.40 ± 2.966	0.325
Direct Bilirubin (mg/dL)	3.83 ± 2.911	4.44 ± 3.170	5.19 ± 3.718	0.119
Total Protein (g/dL)	7.11 ± 3.263	6.59 ± 1.372	6.17 ± 1.095	0.139
Alkaline Phosphatase (U/L)	128.39 ± 65.319	146.72 ± 71.358	136.50 ± 65.125	0.239
Alanine Transaminase (IU/L)	32.65 ± 20.385	33.33 ± 18.781	43.38 ± 32.737	0.064
Aspartate Aminotransferase (IU/L)	71.74 ± 53.591	77.54 ± 46.793	102.87 ± 58.883	0.020*
Globulin (g/dL)	3.80 ± 0.863	3.88 ± 0.693	3.79 ± 1.099	0.801
Albumin (g/dL)	2.69 ± 0.529	2.55 ± 0.531	2.34 ± 0.456	0.006*
**RFT and serum electrolytes** (Mean ± SD)				
Potassium (mmol/L)	4.74 ± 0.698	4.56 ± 1.018	4.74 ± 1.578	0.473
Sodium (mmol/L)	130.31 ± 5.973	130.14 ± 6.260	130.80 ± 7.797	0.891
Creatinine (mg/dL)	1.83 ± 0.874	2.13 ± 0.853	2.36 ± 1.21	0.018*
Serum urea (mg/dL)	51.30 ± 23.53	53.95 ± 25.223	63.40 ± 29.949	0.085
**Coagulation Test** (Mean ± SD)				
INR	1.55 ± 0.418	1.66 ± 0.541	1.76 ± 0.725	0.138
Prothrombin time	16.74 ± 3.951	18.38 ± 5.823	18.95 ± 6.977	0.068

*Significant.

Note: All the Laboratory values are from the first day of AKI diagnosis.

Abbreviations: EV: Esophageal varices; HE: Hepatic encephalopathy; INR: International Normalized Ratio; LFT: Liver Functioning Test; RFT: renal function test: SBP: Spontaneous Bacterial Peritonitis.

**Table 3.** uNGAL Level in AKI type.

AKI Type	PRA Mean ± SD	HRS Mean ± SD	ATN Mean ± SD	ANOVA *p*-value	AKI type	*p*-value
uNGAL (ng/ml)	31.15 ± 18.079	318 ± 104.497	724.03 ± 80.067	0.001	PRA vs. HRS	<0.001*
					PRA vs. ATN	<0.001*
					HRS vs. ATN	<0.001*

*Significant.

Abbreviations: AKI: Acute kidney disease; PRA: Prerenal AKI; HRS: Hepatorenal syndrome; ATN: Acute tubular necrosis; uNGAL: Urinary Neutrophil Gelatinase-Associated Lipocalin.

### uNGAL in the differential diagnosis of AKI

**Figure 3.**
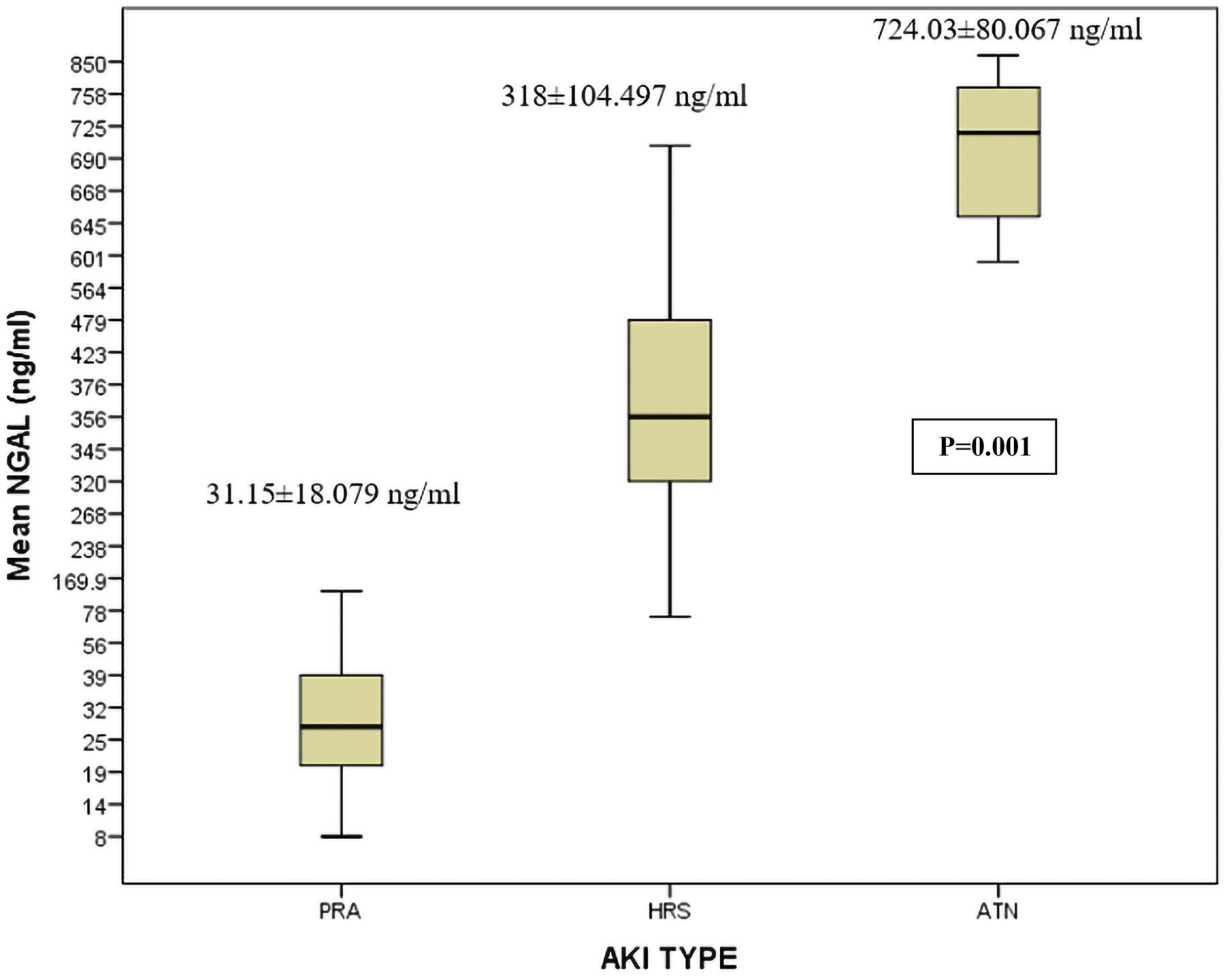
Boxplot for urinary NGAL in different types of AKI.

Urinary NGAL levels showed a clear and statistically significant difference among the three AKI types in cirrhotic patients. The lowest mean value was observed in the PRA group (31.15 ± 18.08 ng/ml), followed by HRS group (318 ± 104.50 ng/ml), and the highest concentration in the TN) group (724.03 ± 80.07 ng/ml) (*p* = 0.001), and post-hoc analysis confirmed that each pairwise comparison was statistically significant (PRA vs. HRS, *p* < 0.001; PRA vs. ATN, *p* < 0.001; HRS vs. ATN, *p* < 0.001) (Table 3 and Figure 3).

**Figure 4.**
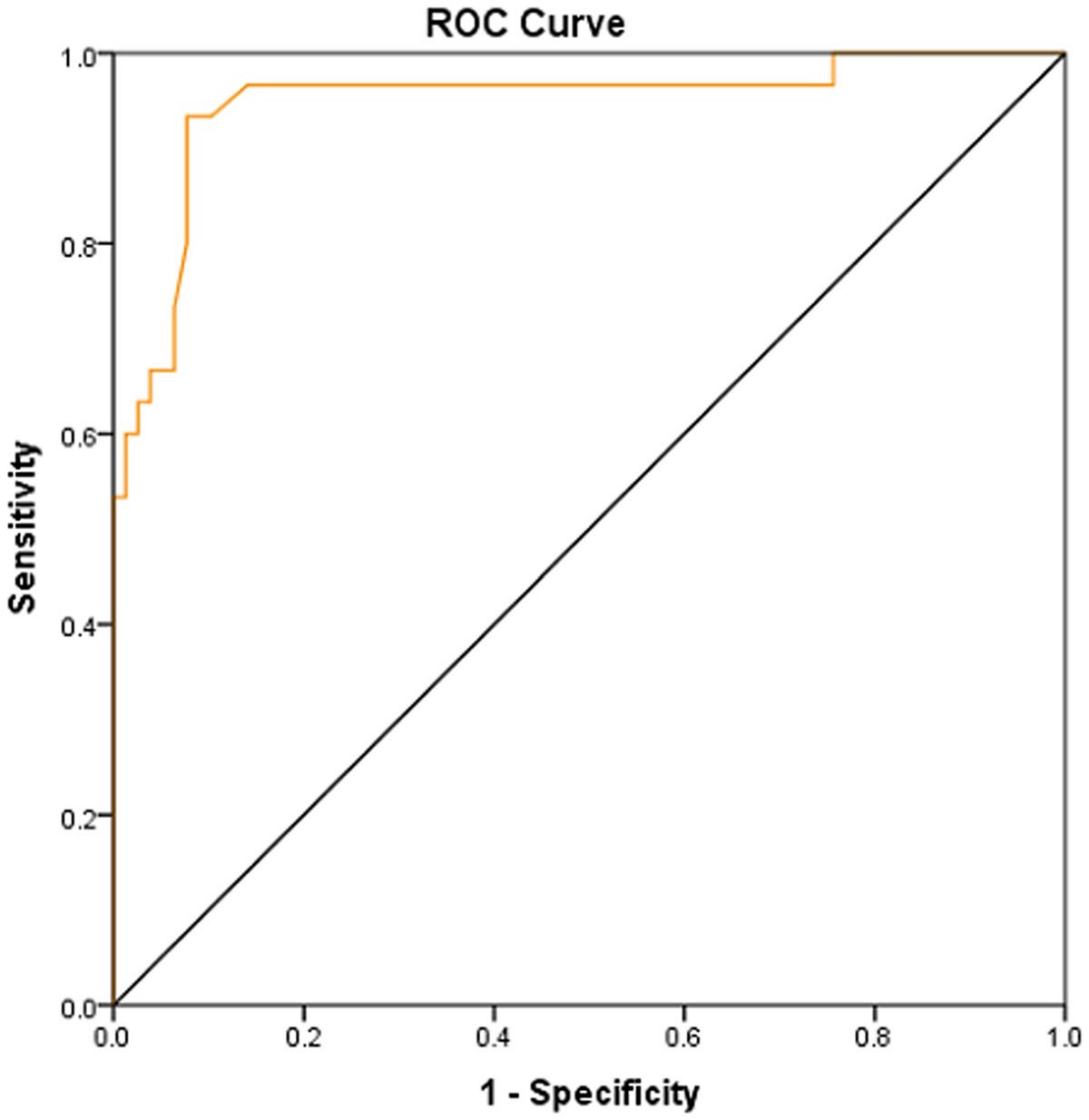
ROC analysis of urinary NGAL (HRS v/s ATN).

The cutoff value for uNGAL distinguishing between HRS and ATN was determined to be 631 ng/ml [AUC: 0.949, 95% C.I (0.895-1)], with a sensitivity of 92% and specificity of 93% (Figure 4).

### Clinical outcome

Clinical outcomes were assessed in terms of mortality, with an overall in-hospital mortality rate of 5.9% (*n* = 11) observed. Furthermore, mortality rates at 30 days and 90 days were recorded at 32.4% (*n* = 61) and 48.9% (*n* = 92), respectively. When considering the type of AKI, ATN demonstrates a higher mortality rate in comparison to PRA and HRS across in-hospital, 30-day, and 90-day timeframes (*P* value < 0.005) (Table 4).

**Table 4.** Clinical outcomes.

Parameters	Overall (*n* = 188)	Prerenal AKI (*n* = 80)	HRS (*n* = 78)	ATN (*n* = 30)	*p*-value
In-hospital Mortality, % (n)	5.9% (11)	1.2% (1)	5.1% (4)	20% (6)	p = 0.001*
30-day mortality, % (n)	32.4% (61)	8.8% (7)	39.7% (31)	76.7% (23)	0.002*
90-day mortality, % (n)	48.9% (92)	17.5% (14)	65.4% (51)	90% (27)	0.001*

*Significant.

Abbreviations: HRS: Hepatorenal syndrome; ATN: Acute tubular.

**Figure 5.**
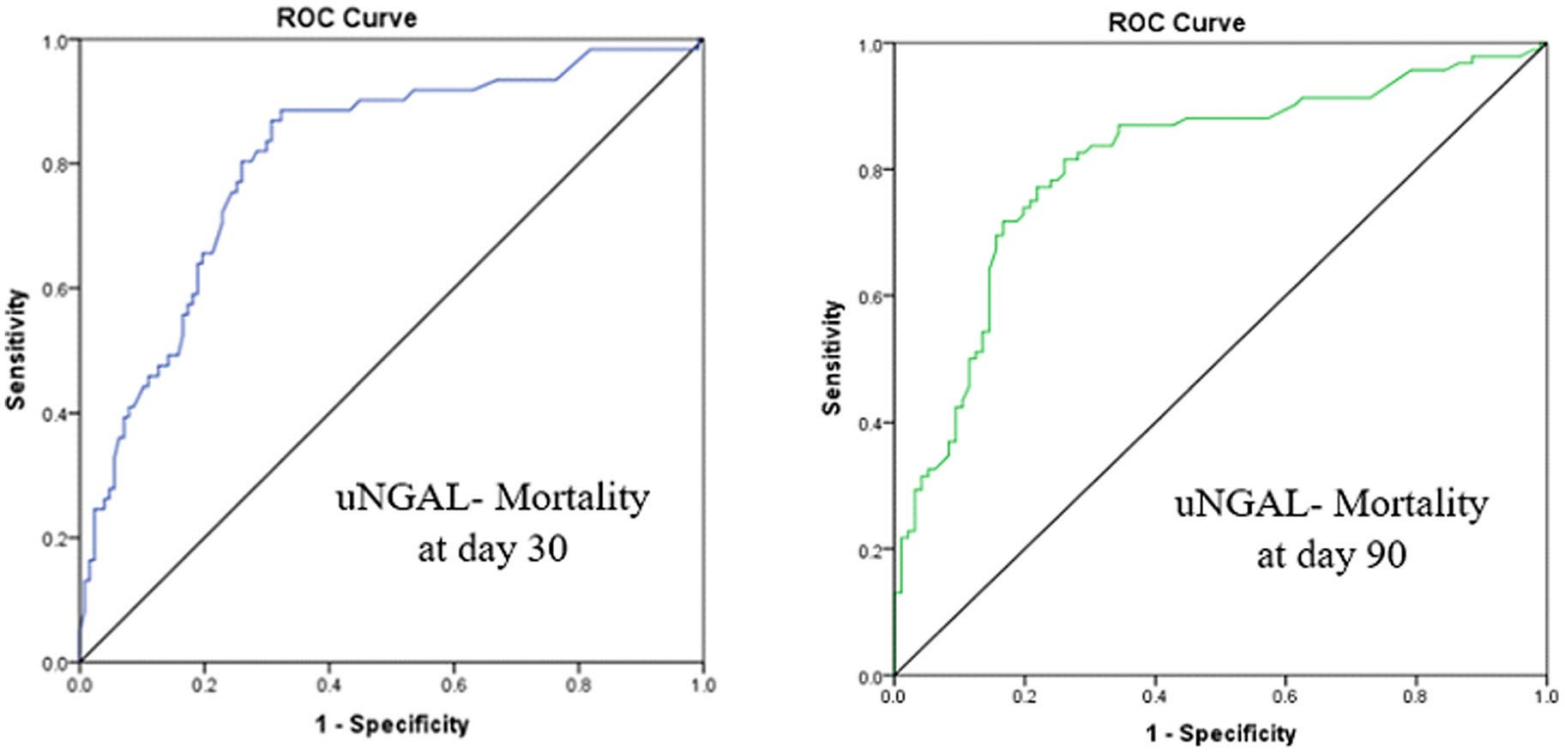
ROC of uNGAL for mortality at day 30 and 90.

For 30-day mortality, uNGAL demonstrated an AUC of 0.807 (95% CI: 0.740–0.875). At the optimal cutoff of 257 ng/mL, uNGAL showed 67% sensitivity and 88% specificity, corresponding to a PPV of 72.8% and NPV of 84.8%, indicating that elevated uNGAL reliably identifies patients at higher risk while also effectively ruling out low-risk cases. For 90-day mortality, uNGAL similarly performed well (AUC 0.809, 95% CI: 0.745–0.873) with a cutoff of 236 ng/mL, yielding 74% sensitivity, 81% specificity, PPV of 78.8%, and NPV of 76.5% (Table 5 and Figure 5).

### Predictors of mortality

Univariate analysis showed that higher AST, Alanine Transaminase (ALT), Alkaline Phosphatase, Globulin, Total Bilirubin, Direct Bilirubin, MELD-sodium (MELD-Na), CTP, Prothrombin Time, International Normalized Ratio, uNGAL, and low sodium are the risk factors for mortality in the study population. Multivariate regression analysis revealed that the elevated ALT, MELD-Na score, and u-NGAL levels were independent risk factors for mortality in the study population (Table 5).

**Table 5.** Risk factors of mortality.

Variable	Univariate analysis		Multivariate analysis	
	Unadjusted-OR (95% CI)	*p*-value	Adjusted-OR (95% CI)	*p*-value
Alkaline Aspartate (U/L)	1.006 (1.00-1.012)	0.050*		
Alanine Transaminase (U/L)	1.017 (1.003-1.032)	0.015*	1.034 (1.005-1.064)	0.022**
Alkaline Phosphatase (U/L)	1.005 (1.00-1.010)	0.050*		
Globulin (g/dL)	1.384 (0.969-1.977)	0.074*		
Sodium (mmol/L)	0.954 (0.91-0.99)	0.047*		
Total bilirubin (mg/dL)	1.162 (1.04-1.28)	0.005*		
Direct bilirubin (mg/dL)	1.133 (1.03- 1.24)	0.010*		
MELD-Na	1.173 (1.11-1.23)	< 0.001	1.117 (1.014-1.232)	0.025**
CTP	1.605 (1.32-1.94)	< 0.001		
Prothrombin Time	1.080 (1.018-1.146)	0.011*		
International Normalized Ratio	1.818 (1.015-3.24)	0.44		
uNGAL (ng/ml)	1.005 (1.004-1.007)	< 0.001	1.005 (1.003-1.007)	p = 0.001**

*Significant (*p* < 0.20), ** Significant (*p* < 0.05).

Abbreviations: CTP: Child-Turcotte-Pugh; MELD-Na: Model for End-Stage Liver Disease- Sodium; OR: Odds Ratio; uNGAL: Urinary Neutrophil Gelatinase-Associated Lipocalin.

**Figure 6.**
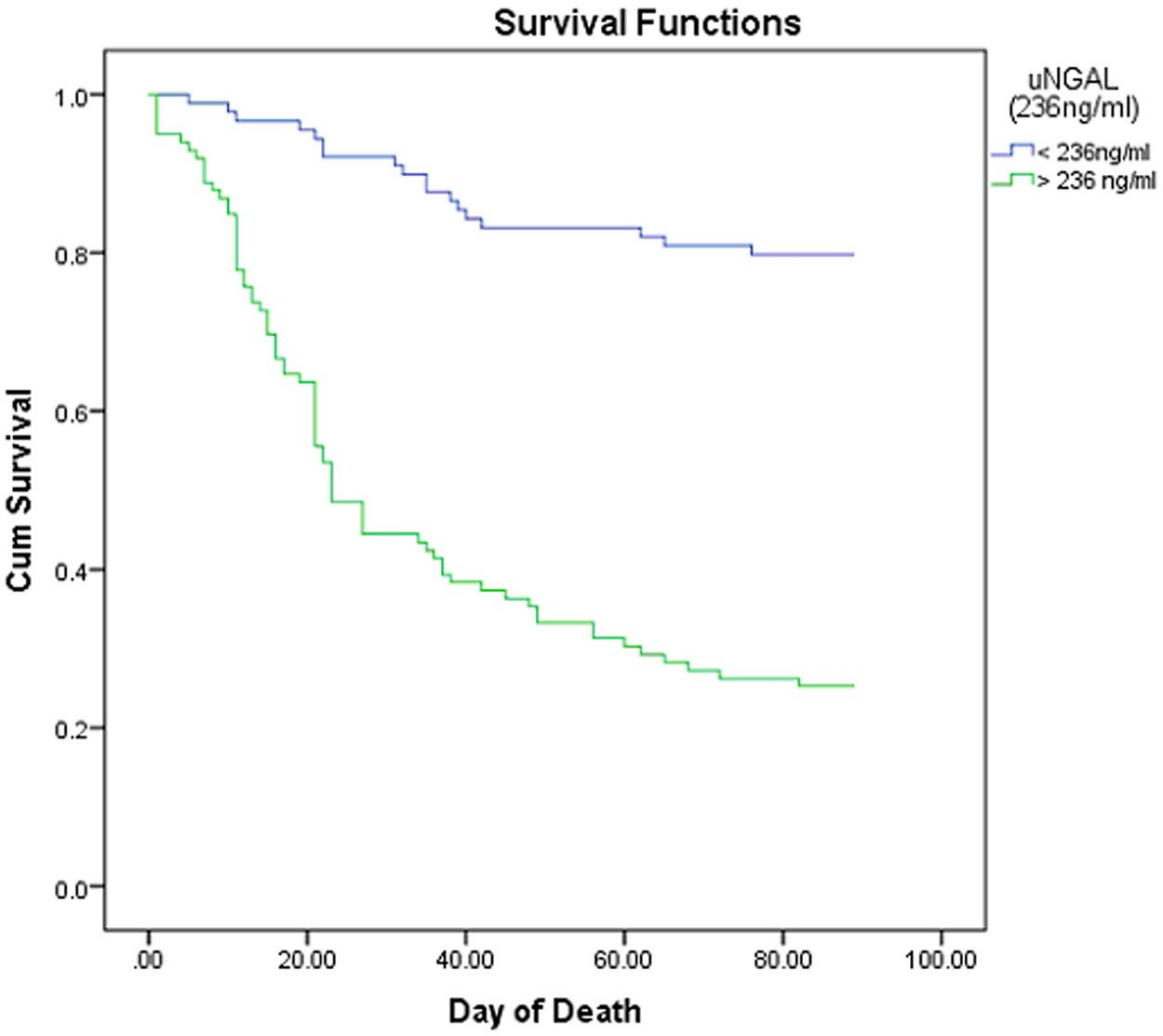
Survival analysis.

The survival curves revealed a clear distinction in 90-day survival outcomes based on uNGAL levels. Patients with uNGAL levels exceeding 236 ng/mL had a significantly higher mortality rate compared to those with levels below this threshold (Figure 6).

## Discussion

In the current clinical practice era, distinguishing HRS from ATN carries significant therapeutic implications. Given the considerable clinical overlap between these two syndromes, many practitioners face challenges in making an accurate diagnosis. An objective diagnostic test, when used alongside guideline-based approaches to managing AKI in cirrhosis, can enhance both the speed and precision of diagnosis [5,9]. The typical forms of non-HRS-AKI include PRA, ATN majorly [25].

In the present study of the 188 patients, 42.6% (*n* = 80) exhibited prerenal AKI, 41.5% (*n*= 78) were diagnosed with HRS, and 16% (*n* = 30) were identified as having ATN. In the study conducted by Moreau et al., the most common causes of AKI in cirrhotic patients were PRA, observed in 49% of cases, and ATN, which accounted for 35% of the cases. A study from South India revealed that prerenal AKI incidence accounted for a significant portion of AKI cases, with 37.5% (*n* = 75) classified as prerenal AKI, 34% (*n* = 68) as HRS-AKI, and 28.5% (*n* = 57) as ATN [26]. In a large, multicenter cohort of 1,456 patients with cirrhosis and AKI, Patidar et al. reported that hypovolemic AKI was the most common phenotype (58.9%), followed by HRS-AKI (17.4%) and ATN (14.8%) [27].

In terms of AKI staging indicated that 52.1% (*n* = 98) were categorized as AKI stage 1, 39.9% (*n* = 75) as AKI stage 2, and only 8% (*n* = 15) as AKI stage 3. Likewise, a similar pattern of grading was observed by Khatua et al. [28] where out of 315 individuals with AKI, 34% (*n* = 106) were classified as stage 1 A, 21% (*n* = 65) as stage 2, and 17% (*n* = 54) as stage 3 AKI.

The severity scores for the diseases, MELD, MELD Na, and CTP, showed a notable rise in the ATN group, followed by increases in the HRS and PRA groups, with statistical significance observed (*p* = 0.001). Within the CTP C group, 52.7% were categorized, with HRS representing 45.5% (*n* = 45), a proportion significantly higher compared to both the ATN and PRA groups (*p* = 0.002).

In the present study, mean ± SD uNGAL for PRA is 31.15 ± 18.079 ng/ml, HRS is 318 ± 104.497 ng/ml and ATN is 724.03 ± 80.067 ng/ml. Our study confirmed that uNGAL was significantly higher in ATN compared to HRS and ATN. A cutoff value of 631 ng/ml for uNGAL was established to effectively differentiate between HRS and ATN, exhibiting an AUC of 0.949 (95% C.I 0.895–1), with a sensitivity of 92% and specificity of 93%.

In a study by Treeprasertsuk.et al. [29] a similar trend was observed, with higher mean uNGAL levels detected in patients with ATN compared to those with HRS and PRA, respectively ‘(639.7 ± 532.2 vs. 241.0 ± 41.0 vs. 163.8 ± 156.2 ng/mL, *p* = 0.001)’. The optimal uNGAL cutoff value of 136.8 ng/mL yielded a sensitivity of 88.9% and specificity of 80.4% for diagnosing ATN, with an AUC-ROC of 0.91 (95% CI: 0.83–0.98, *p* < 0.05). Michael et al. 2025 reported that uNGAL effectively differentiates intrinsic AKI from non-intrinsic AKI (pre- and post-renal). Intrinsic AKI patients had markedly higher uNGAL levels than non-intrinsic AKI (1052.4 vs. 228.2 ng/mL; *p* < 0.001) [30]. Sharan et al. reported that uNGAL was significantly higher in ATN (1747 [6–6141] ng/mL) compared to HRS (379 [33.5–2320] ng/mL; *p* < 0.0001) and PRA (167 [3.34–660] ng/mL; *p* < 0.0001) [31].

Another study also found that PRA had lower median uNGAL values compared to HRS and ATN (95.50 *vs* 465.00 *vs* 1217.50, *p* < 0.001). Qaseem et al. [32] used uNGAL cut‐off values of 286.3 𝜇g/g creatinine (AUROC: 0.909) to differentiate HRS from ATN. The diagnostic accuracy of uNGAL for distinguishing between HRS and ATN was found to be high, with an AUROC of 0.94. The most effective cutoff value was determined to be 650 ng/mL, which yielded a sensitivity of 100% and a specificity of 83.78%. Based on this threshold, uNGAL levels below 650 ng/mL are more consistent with a diagnosis of HRS, whereas levels above 650 ng/mL are more indicative of ATN. These findings support the utility of uNGAL as a noninvasive biomarker for differentiating between these two clinically overlapping but pathophysiologically distinct forms of AKI in cirrhotic patients [33].

An overall in-hospital mortality rate of 5.9% (*n* = 11) was observed. Furthermore, mortality rates at 30 days and 90 days were recorded at 32.4% (*n* = 61) and 48.9% (*n* = 92), respectively. Across in-hospital, 30-day, and 90-day periods, ATN demonstrated a higher mortality rate compared to PRA and HRS. Musunuri et al. [34] reported a high in-hospital mortality rate in their study (28.4%), with a 90-day mortality of 39.21%. Regarding AKI type, the HRS group exhibited a high 90-day mortality rate of 54.54%. A recent study Patidar et al. [35], demonstrated that patients with PRA had the lowest mortality rate, whereas mortality was higher in those with ATN at 52.7%; however, this was not significantly different from the mortality rate observed in patients with HRS (49.0%).

In multivariate analysis, higher ALT, MELDNa and uNGAL were independent risk factors for mortality in the study population. Gameiro et al. [36] recognized MELDNa as independent predictive factors for AKI. Another similar study by Allegretti et al. demonstrated that a higher MELD score and elevated sCr, serum albumin and total bilirubin were significantly associated with 90-day mortality (*p* < 0.05) [10].

Lee et al. found that uNGAL was an independent risk factor for mortality with AKI [37]. In a multicenter prospective study conducted by Kim et al. 2020 uNGAL and cystatin C were investigated in 328 cirrhosis patients (AKI = 41) [38]. The study revealed that uNGAL serves as a predictor for both AKI and mortality. Barreto et al. 2014 in their univariate analysis, found that uNGAL, MELD score, and length of hospital stay were statistically significant factors differentiating survivors from non-survivors, indicating their potential value as prognostic indicators in patients with cirrhosis and AKI [39]. Similarly, Verna et al. 2012 reported that, on univariate analysis, both uNGAL and MELD score were predictive of poor clinical outcomes [40].

In our study, uNGAL values >236ng/ml had a shorter survival time compared with those with uNGAL levels <236ng/ml. Similarly, Udgirkar S et al. 2020 (33) reported that patients with higher NGAL levels (>289.6 μg/dL) experienced significantly reduced survival compared to those with lower levels (<289.6 μg/dL). Their findings are further supported by Gungor et al. 2014 who also demonstrated that elevated NGAL is associated with poorer outcomes [41]. The present study highlighted the role of uNGAL levels in the differential diagnosis of AKI; however, it has certain limitations, including being conducted at a single center, having a relatively small sample size, reliance on the 2015 ICA-AKI definition, and assessing NGAL at a single time point without follow-up measurements. The 90-day mortality observed for ATN in our cohort was higher than reported in most studies, likely due to a selection bias toward more severe cases, which may have amplified the mortality differences between ATN and HRS. Additionally, NGAL shows relatively low sensitivity but higher specificity, limiting its effectiveness as a screening tool for 30-day mortality. Therefore, further validation using updated criteria and replication in larger, multicenter cohorts are necessary to confirm these results.

## Conclusion

The study’s findings indicate significant variations in uNGAL levels among different types of AKI, with the lowest in patients with PRA and the highest in those with ATN. The determined cutoff value for uNGAL to differentiate between HRS and ATN was 631 ng/mL, demonstrating high sensitivity and specificity. Furthermore, uNGAL proved to be a valuable biomarker for predicting 30-day and 90-day mortality, with respective cutoff values of 257 and 236 ng/ml providing robust predictive capabilities. Clinical outcomes indicate a higher mortality rate in ATN compared to PRA and HRS. In addition, higher levels of ALT, MELD-Na, and uNGAL as independent risk factors for mortality.

## Data Availability

The datasets generated and/or analyzed during the current study are available from the corresponding author on reasonable request.
